# Role of silicon in alleviating boron toxicity and enhancing growth and physiological traits in hydroponically cultivated *Zea mays* var. Merit

**DOI:** 10.1186/s12870-024-05275-2

**Published:** 2024-06-14

**Authors:** Farhad Behtash, Farima Mogheri, Ahmad Aghaee, Hanifeh Seyed Hajizadeh, Ozkan Kaya

**Affiliations:** 1 Department of Horticulture, Faculty of Agriculture, University of Maragheh, Maragheh, 55136-553 Iran; 2 Department of Biology, Faculty of Science, University of Maragheh, Maragheh, Iran; 3 Erzincan Horticultural Research Institute, Republic of Turkey Ministry of Agriculture and Forestry, Erzincan, 24060 Turkey; 4 Department of Plant Sciences, North Dakota State University, Fargo, ND 58102 USA

**Keywords:** Antioxidant capacity, Boron exceeds, Minerals, Sweet corn, Silicon, Performance

## Abstract

**Background:**

Boron (B) is a micronutrient, but excessive levels can cause phytotoxicity, impaired growth, and reduced photosynthesis. B toxicity arises from over-fertilization, high soil B levels, or irrigation with B-rich water. Conversely, silicon (Si) is recognized as an element that mitigates stress and alleviates the toxic effects of certain nutrients. In this study, to evaluate the effect of different concentrations of Si on maize under boron stress conditions, a factorial experiment based on a randomized complete block design was conducted with three replications in a hydroponic system. The experiment utilized a nutrient solution for maize var. Merit that contained three different boron (B) concentrations (0.5, 2, and 4 mg L^−1^) and three Si concentrations (0, 28, and 56 mg L^−1^).

**Results:**

Our findings unveiled that exogenous application of B resulted in a substantial escalation of B concentration in maize leaves. Furthermore, B exposure elicited a significant diminution in fresh and dry plant biomass, chlorophyll index, chlorophyll a (Chl a), chlorophyll b (Chl b), carotenoids, and membrane stability index (MSI). As the B concentration augmented, malondialdehyde (MDA) content and catalase (CAT) enzyme activity exhibited a concomitant increment. Conversely, the supplementation of Si facilitated an amelioration in plant fresh and dry weight, total carbohydrate, and total soluble protein. Moreover, the elevated activity of antioxidant enzymes culminated in a decrement in hydrogen peroxide (H_2_O_2_) and MDA content. In addition, the combined influence of Si and B had a statistically significant impact on the leaf chlorophyll index, total chlorophyll (a + b) content, Si and B accumulation levels, as well as the enzymatic activities of guaiacol peroxidase (GPX), ascorbate peroxidase (APX), and H_2_O_2_ levels. These unique findings indicated the detrimental impact of B toxicity on various physiological and biochemical attributes of maize, while highlighting the potential of Si supplementation in mitigating the deleterious effects through modulation of antioxidant machinery and biomolecule synthesis.

**Conclusions:**

This study highlights the potential of Si supplementation in alleviating the deleterious effects of B toxicity in maize. Increased Si consumption mitigated chlorophyll degradation under B toxicity, but it also caused a significant reduction in the concentrations of essential micronutrients iron (Fe), copper (Cu), and zinc (Zn). While Si supplementation shows promise in counteracting B toxicity, the observed decrease in Fe, Cu, and Zn concentrations warrants further investigation to optimize this approach and maintain overall plant nutritional status.

## Background

*Zea mays* L., commonly referred to as maize or corn, is a predominant cereal crop that holds a pivotal position in global agricultural systems. Among cereals, maize exhibits the highest levels of cultivation area, production, and yield, with 1397 million hectares under cultivation, a production of 1.137 million metric tons, and an average yield of 5.8 metric tons per hectare as of 2019 [1]. Maize contributes significantly to agri-food systems worldwide, and the human food pathway based on maize plays a pivotal role in enhancing food security and the nutritional status of vulnerable populations. However, crop production is frequently constrained by excessive concentrations of mineral nutrients in plants growing media. Agricultural soils often exhibit toxic levels of certain minerals, including iron (Fe), chloride (Cl), sodium (Na), boron (B), manganese (Mn), and aluminum (Al) [2]. Substantial variations in boron concentrations have been detected across different soil textures, with some soils containing excessive boron levels that trigger phytotoxicity, while others exhibit inadequate B concentrations to support normal plant growth [3]. In plant leaf tissues, B typically exists at concentrations ranging from 10 to 50 mg kg^−1^. Numerous crop species, particularly major cereals such as maize and wheat, exhibit high sensitivity to elevated boron levels, displaying toxicity symptoms in their tissues [4]. Boron toxicity induces deleterious effects on the physiological and morphological characteristics of plants, including reduced shoot and root growth, impaired root cell division, increased root lignin and suberin deposition, decreased stomatal conductance, inhibited photosynthesis, increased membrane permeability, lipid peroxidation, and elevated antioxidant enzyme activities [3, 5]. Corn is considered moderately sensitive to boron (B) toxicity in actual production conditions. While corn requires B for proper growth and development, it can exhibit adverse effects when exposed to excessive B levels in the soil or irrigation water [3, 4]. Boron toxicity in corn can lead to reduced yields, stunted plant growth, and leaf necrosis, making it a significant concern in areas with high soil B concentrations or B-rich irrigation sources [4]. Consequently, boron toxicity elicits oxidative stress in plant cells, resulting in the production of reactive oxygen species (ROS) such as hydrogen peroxide (H_2_O_2_), superoxide radical (O^2−^), and hydroxyl radical (^•^OH) [3, 6]. The accumulation of ROS leads to progressive oxidative damage to proteins, lipids, and nucleic acids, ultimately causing lipid peroxidation, cell membrane damage, enzyme inactivation, and cell death [7].

Silicon is, on the other hand, considered a beneficial element for plants, even though it is not classified as an essential nutrient. Silicon has been shown to enhance plant tolerance to various abiotic stresses such as drought, salinity, metal toxicity, and disease resistance [8]. Application of silicon fertilizers can improve plant growth, yield, and biomass production in many crops, especially cereals like rice, wheat, and maize. In addition, it helps strengthen cell walls by depositing them as silica gel, providing structural rigidity and preventing lodging in cereal crops. It has been demonstrated to improve growth and yield in cereals and legumes, as well as enhance drought tolerance in plants [9]. Furthermore, the crucial role of silicon in increasing chlorophyll content and promoting photosynthesis, growth, and productivity in cucumber plants has been documented [10]. The reduction of lipid peroxidation in silicon-treated plants has been attributed to the strengthening of resistance mechanisms in these plants [11]. It has been described that silicon decreases the content of H_2_O_2_ and MDA by increasing the activity of antioxidant enzymes, such as catalase and peroxidase [8]. Additionally, silicon has been reported to ameliorate metal toxicities, including Al, Cd, and Mn, in several plant species [12]. Silicon has also been found to alleviate boron toxicity and enhance boron tolerance in some plant species, such as wheat and tomato [13, 14]. The pivotal mechanisms underpinning the silicon-mediated mitigation of boron toxicity encompass the diminution of heavy metal concentrations in the plant growth milieu, attenuated boron uptake and root-to-shoot translocation, complexation, and co-precipitation of boron with silicon, and the stimulation of antioxidant systems within the plants [12, 13]. Considering the potential effects of silicon on stimulating plant growth, productivity, and improving tolerance to toxicities, this study was conducted to elucidate the effectiveness of silicon in developing tolerance mechanisms in maize var. Merit in response to boron toxicity. In this regard, various growth, and physiological indices, as well as their antioxidative capacity, were evaluated.

## Results and discussion

### Physiological traits

**Fig. 1:**
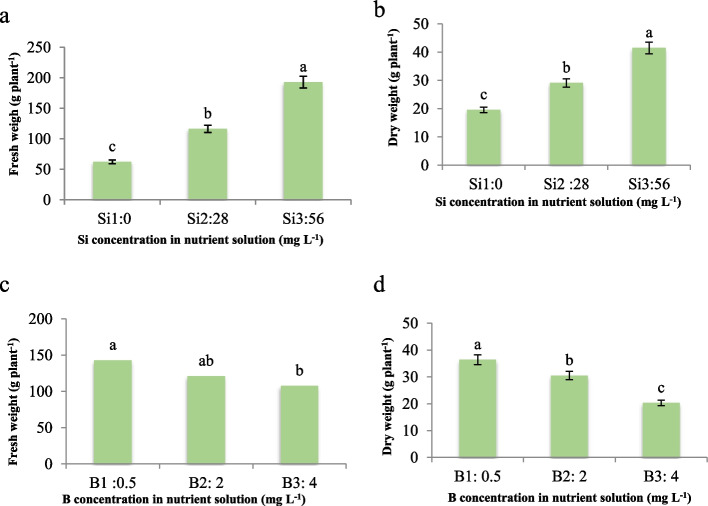
Effect of different concentrations Si on **a**) fresh weight and **b**) dry weight and different concentrations of B on **c**) fresh weight and **d**) dry weight of maize var. Merit. Means not sharing the same letter do not differ significantly at *p *≤ 0.01

Considering fresh weight/dry weight, our findings indicate that increasing silicon (Si) concentration in the nutrient solution led to a substantial increase in both fresh and dry weight of maize plants, with fresh weight increasing up to threefold and dry weight increasing up to twofold (Figs. 1a and b). Conversely, boron (B) exhibited a detrimental effect, causing a reduction in fresh and dry weight. Notably, the impact of B on decreasing dry weight (44%) was more pronounced compared to its effect on fresh weight (24%) when compared to the control plants (Figs. 1c and d). These results are consistent with previous studies that have demonstrated the beneficial effects of silicon supplementation on plant growth and development [13, 14]. Although silicon is not considered an essential element for plants, numerous reports have proven its efficacy in promoting optimal growth and development across various plant species [8]. Several studies have documented the positive impact of silicon application on increasing yield and leaf area in plants. Our findings align with the results obtained by Zhu et al. [10] in cucumber, where silicon supplementation enhanced growth parameters. Furthermore, under heavy metal stress conditions, increased silicon levels have been shown to mitigate the deleterious effects of cadmium and boron in maize [15] and wheat [13]. Based on our results, we hypothesize that the underlying mechanism contributing to the observed growth promotion by silicon may be attributed to its deposition as amorphous silica in cell walls. This deposition process is, indeed, assumed to protect plant cells against abiotic stresses, such as boron toxicity, and stabilize cell wall structures, thereby facilitating enhanced vegetative growth [16]. The capacity of silicon to alleviate boron toxicity and promote growth can be ascribed to its pivotal role in fortifying antioxidant defense mechanisms, mitigating oxidative stress, and preserving cellular integrity under stress-induced conditions. On the other hand, although B is considered a microelement, its excessive amounts in nutrient solution will lead to pepper toxicity [17]. Reduced vegetative growth and yield under B toxicity conditions have been proven in many plants [13] as its toxicity is more effective than its deficiency in plant performance [3].

**Fig. 2:**
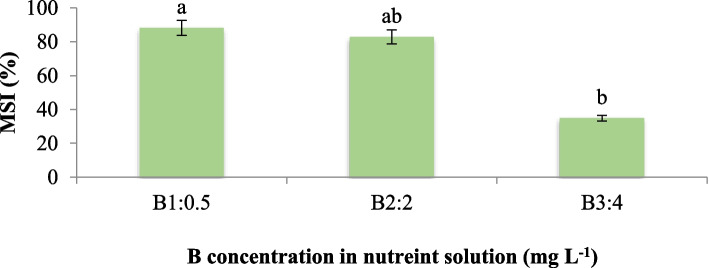
Effect of different concentrations of B on leaf MSI of maize var. Merit**.** Means not sharing the same letter do not differ significantly at *p* ≤ 0.01

Our findings indicate that an increase in boron (B) concentration in the nutrient solution adversely affected the stability of leaf membranes, as evidenced by a significant decrease of 60.5% in the membrane stability index (MSI) (Fig. 2). Notably, neither silicon (Si) supplementation alone nor the interaction effect of Si and B had a significant impact on MSI. We hypothesize that the underlying mechanism contributing to the observed decrease in membrane stability may be attributed to the peroxidation of membrane lipids induced by the activity of free radicals. These results are consistent with previous studies that have reported an increase in electrolyte leakage and a consequent decline in membrane stability due to boron toxicity in various plant species, including onion, tomato, cucumber, sorghum, and maize [18, 19]. Under stress conditions, one of the effects of accumulated free oxygen species in plant cells is the peroxidation of phospholipids, which is accompanied by the oxidation of unsaturated fatty acids. This process is assumed to cause membrane destruction and subsequent electrolyte leakage [20]. The oxidative damage to membrane lipids and the resulting loss of membrane integrity could be a key reason for the reduced membrane stability observed in our study under boron toxicity. In addition, our findings are consistent with previous studies that have reported a significant impact of boron toxicity on membrane stability and electrolyte leakage in various plant species [18, 19]. The underlying mechanism proposed is the peroxidation of membrane lipids by free radicals, leading to oxidative damage and compromised membrane integrity. This hypothesis aligns with the well-established role of reactive oxygen species in inducing oxidative stress and lipid peroxidation under stress conditions, ultimately resulting in membrane destabilization and electrolyte leakage [11].

**Fig. 3:**
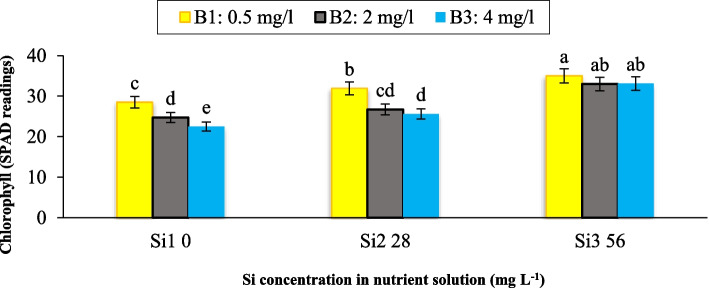
Interaction effect of different concentrations of Si and B on leaf chlorophyll of maize var. Merit. Means not sharing the same letter do not differ significantly at *p* ≤ 0.01

Our findings indicate a significant interaction effect (*p* ≤ 0.01) between Si and B on leaf chlorophyll content. The maximum leaf chlorophyll level was observed in plants treated with 0.5 mg L^−1^ of B and 56 mg L^−1^ of Si, while the minimum value was recorded in plants exposed to 4 mg L^−1^ of B without Si supplementation. Mean comparisons revealed that as Si concentration in the nutrient solution increased, chlorophyll content also increased from 28.5 in the control plants to 31.9 and 35.0 in plants treated with 0.5 mg L^−1^ B + 28 mg L^−1^ Si and 0.5 mg L^−1^ B + 56 mg L^−1^ Si, respectively, with significant differences compared to the control (Fig. 3). These results indicate that chlorophyll content generally decreased with increasing B concentration in the nutrient solution, but this effect was modulated by Si application. Notably, the maximum Si concentration (56 mg L^−1^) was more effective in mitigating the adverse effects of B, even at the highest B concentration tested (4 mg L^−1^). The application of Si, both under B toxicity conditions and in the absence of B, caused an increase in chlorophyll content compared to other treatments (Fig. 3). These findings suggest that Si plays a protective role in preventing the destruction and reduction of chlorophyll caused by B toxicity. Our findings are consistent with previous studies, such as the work of Erslan et al. [17] in pepper and tomato, which reported similar trends. Al-Agha Bary et al. [21] reported that Si application reduced the production of H_2_O_2_ under stress conditions and increased leaf chlorophyll content in tomatoes. In addition, we hypothesize that one of the underlying mechanisms contributing to the positive effect of Si on chlorophyll content may be related to its ability to enhance the activity of photosystem II. Several studies have suggested that silicon strengthens mature leaves, increases the amount of chlorophyll per leaf area, and prevents leaf senescence [22]. This protective role of Si in maintaining chlorophyll levels and delaying leaf senescence could be attributed to its capacity to alleviate oxidative stress and improve the overall photosynthetic efficiency of plants under stress conditions.

**Fig. 4:**
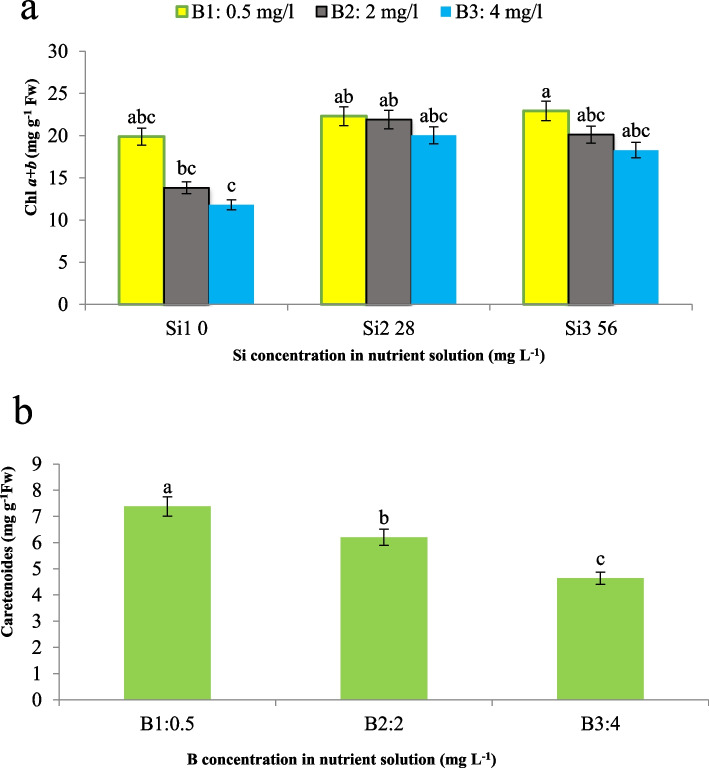
Interaction effect of different concentrations of Si and B on **a**) Chl *a* + *b* and the effect of B concentration on **b**) carotenoids of maize var. Merit. Means not sharing the same letter do not differ significantly at *p* ≤ 0.01

Our findings reveal a similar trend in the levels of Chl *a*, Chl *b*, total chlorophyll (Chl a + b), and carotenoids, where the maximum Chl a + b content was observed in plants treated with 0.5 mg L^−1^ of B and 56 mg L^−1^ of Si, while the minimum value was recorded in plants exposed to 4 mg L^−1^ of B without Si supplementation. According to Fig. 4a, the effect of Si in mitigating the adverse effects of B was more pronounced at medium Si concentrations compared to higher concentrations. These results are consistent with previous studies that have reported a decrease in photosynthetic pigments, including chlorophyll and carotenoids, under conditions of boron toxicity and other environmental stresses. Leaf senescence, which is typically associated with visible color changes from green to golden yellow, can be accelerated by external factors such as heavy metal stress [23]. In our study, we observed a decrease in maize carotenoid content by 15.9% and 37.1% at B concentrations of 2 and 4 mg L^−1^, respectively. The maximum carotenoid content was found at the lowest B concentration (0.5 mg L^−1^), indicating that increasing B levels resulted in a reduction in carotenoid levels (Fig. 4b). In our findings, we hypothesize that the underlying mechanism contributing to the decrease in photosynthetic pigments under boron toxicity may be related to the generation of different types of free radicals, which can induce oxidative stress and impair the biosynthesis or stability of these pigments. Indeed, B toxicity has been shown to cause a reduction in chlorophyll and other photosynthetic pigments, as well as growth inhibition, with visible symptoms of leaf chlorosis [24]. On the other hand, the decrease in chlorophyll content could be attributed to either the inhibition of its biosynthesis or the degradation of precursor molecules involved in pigment synthesis. It is well-established that oxidative stress, induced by various abiotic stresses, including boron toxicity, can disrupt the biosynthesis and stability of photosynthetic pigments, ultimately leading to their reduction and the manifestation of leaf chlorosis [23, 24]. Our findings also suggest that silicon supplementation can mitigate the adverse effects of boron toxicity on photosynthetic pigments, potentially by enhancing antioxidant defense mechanisms and reducing oxidative stress. Thus, the protective role of silicon in maintaining photosynthetic pigment levels could contribute to improved photosynthetic efficiency and overall plant growth and productivity under stress conditions.

### Biochemical trait

**Fig. 5:**
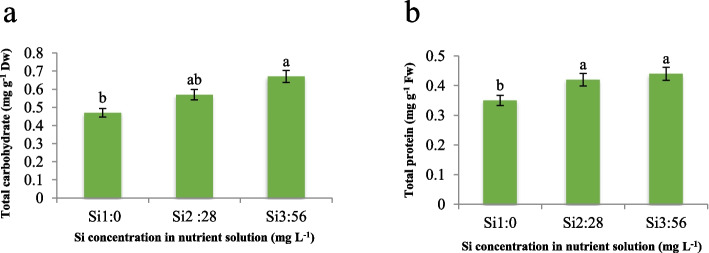
Effect of Si on **a**) total carbohydrate and **b**) total protein of maize var. Merit. Means not sharing the same letter do not differ significantly at *p* ≤ 0.01

Regarding total carbohydrate and total protein, our results demonstrate a substantial 42.5% increase in total carbohydrate (Fig. 5a) and a 25.7% increase in total protein (Fig. 5b) compared to the control treatment. Our findings align with previous studies, indicating a potential underlying mechanism at play [21, 25]. We hypothesize that the observed increase in total carbohydrate and total protein concentrations upon elevated Si supplementation in the nutrient solution is attributed to enhanced photosynthetic efficiency, as reported by numerous researchers. It is plausible that the augmented photosynthetic rate facilitated by Si application leads to an accumulation of carbohydrates within the plant tissues. This phenomenon is consistent with observations of increased total carbohydrate levels under various abiotic stress conditions, such as salinity, drought, low temperature, and heavy metal exposure [25]. Furthermore, the elevation in total protein concentration corroborates findings from previous studies involving tomato [21], cucumber [10], and maize [15], wherein abiotic stresses stimulated an upregulation of protein synthesis. While our results align with the existing literature, the precise mechanistic underpinnings warrant further investigation. Here, we hypothesize that the observed increases in total carbohydrate and total protein concentrations may be attributed to the role of Si in mitigating abiotic stress and optimizing photosynthetic performance, consequently leading to heightened metabolic activity and macromolecular synthesis within the plant system.

**Fig. 6:**
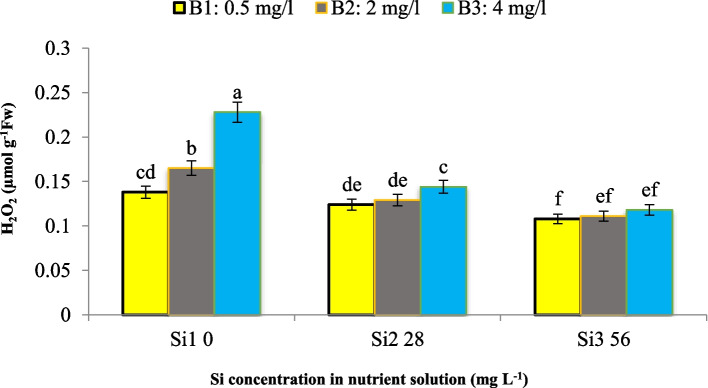
Interaction effect of different concentrations of Si and B on H_2_O_2_ content of maize var. Merit. Means not sharing the same letter do not differ significantly at *p* ≤ 0.01

According to Fig. 6, an increase in B concentration in the nutrient solution led to an elevated H_2_O_2_ content in the leaves, with a 19.5% and 65.2% increase compared to the control and without Si application, respectively. However, the concomitant application of Si mitigated the H_2_O_2_ increment, as evidenced by the lowest H_2_O_2_ content (0.118 µmol g^−1^ FW) observed under severe B stress (4 mg L^−1^) when supplemented with 56 mg L^−1^ Si, in contrast to the elevated H_2_O_2_ level (0.228 µmol g^−1^ FW) under severe B stress without Si application. Our findings corroborate previous studies, indicating a potential underlying mechanism that elucidates the observed trends [13]. Indeed, Si exhibited a positive effect in preventing H_2_O_2_ production even under control conditions without B toxicity, as illustrated in Fig. 6. Our results align with those obtained by Gunes et al. [13]. We also hypothesize that the underlying mechanism behind these observations is related to the accumulation of ROS under B toxicity conditions. B-toxicity favors the accumulation of ROS, such as superoxide radicals (O^2−^), hydroxyl radicals (OH^−^), and H_2_O_2_. Among these, the hydroxyl radical (OH^−^) is the initiator of reactions that lead to lipid peroxidation and accelerated senescence cascades in plants [26]. We assume that the application of Si mitigates the oxidative stress induced by B toxicity, thereby preventing the excessive accumulation of H_2_O_2_ and other ROS. Therefore, this protective effect of Si may be attributed to its role in enhancing the antioxidant defense mechanisms within the plant system, consequently minimizing oxidative damage and maintaining cellular homeostasis.

**Fig. 7:**
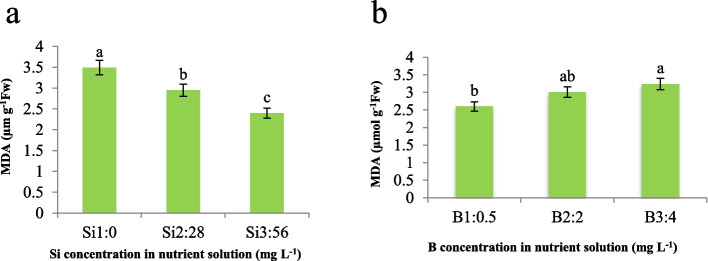
Effect of **a**) Si and **b**) B in nutrient solution on MDA content of maize var. Merit. Means not sharing the same letter do not differ significantly at *p* ≤ 0.01

Our results demonstrate that an increase in Si concentration led to a decrease in MDA content, a byproduct of lipid peroxidation, by 15.4% and 31.2% at 28 and 56 mg L^−1^ Si, respectively (Fig. 7a). Conversely, an increase in B concentration resulted in an elevated MDA content (Fig. 7b). These observations are consistent with the existing literature, which suggests that Si supplementation mitigates MDA accumulation under various abiotic stress conditions, such as heavy metal toxicity, thereby contributing to the maintenance of membrane integrity and enhancing membrane stability [15]. Besides, we assume that the underlying mechanism behind the Si-mediated reduction in MDA content is related to its role in alleviating oxidative stress and strengthening cellular structures. MDA, being a reactive aldehyde, can compromise membrane structure and function by binding to membrane proteins and enzymes. Specifically, MDA can form covalent adducts with amino acid residues in proteins, leading to alterations in their structure and functional properties. This can impair the activity of membrane-bound enzymes involved in vital processes such as ion transport, signal transduction, and metabolic pathways. Additionally, MDA can interact with phospholipids, disrupting the integrity of the lipid bilayer and increasing membrane permeability. Consequently, the compromised membrane structure can lead to leakage of cellular contents, disturbances in ion gradients, and impaired compartmentalization, ultimately hampering cellular homeostasis and function. Silicon application to the plant growth medium is known to reduce the permeability of the leaf cell wall and diminish lipid peroxidation. Additionally, we assume that Si fortifies the cell wall under stressful conditions, thereby protecting it from degradation [13]. This protective effect of Si may be attributed to its ability to enhance the antioxidant defense mechanisms within the plant system, consequently minimizing oxidative damage and preserving membrane integrity. Furthermore, our findings indicate that Si plays a crucial role in mitigating the deleterious effects of B toxicity on membrane lipids. By reducing MDA accumulation, Si may have potentially prevented the propagation of oxidative stress cascades and associated membrane damage, thus contributing to the overall stress tolerance, and maintaining cellular homeostasis of the plant system.

**Fig. 8:**
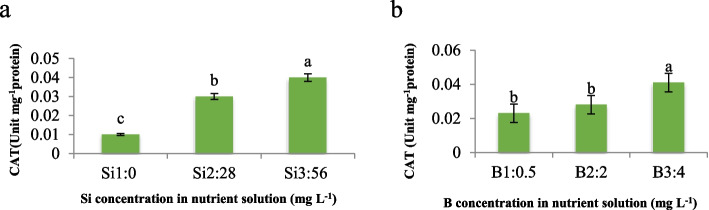
Effect of **a**) Si and **b**) B in nutrient solution on CAT activity of maize var. Merit. Means not sharing the same letter do not differ significantly at *p* ≤ 0.01

Regarding varying concentrations of Si and B on CAT activity in maize plants, we observed a notable trend wherein CAT activity increased proportionally with increasing Si concentrations, peaking at 3- and fourfold increments in CAT activity at Si levels of 28 and 56 mg L^−1^, respectively (Fig. 8a). Similarly, the presence of boron in the nutrient solution exhibited a parallel effect, with CAT activity showing a more pronounced increase at 4 mg L^−1^ compared to 2 mg L^−1^ of B (Fig. 8b). Notably, our findings align with established literature, notably the previously documented B toxicity threshold for maize, which is 4 mg L^−1^. This consistency suggests a robust relationship between Si and B concentrations and CAT activity in maize. Furthermore, our results mirror those observed in cucumber under Mn toxicity conditions, as outlined in previous studies [27]. This consistency across plant species and under differing stressors underscores the reliability and universality of our findings. To elucidate the underlying mechanism driving this observed phenomenon, we propose that Si and B may act synergistically or independently to modulate CAT activity through various physiological pathways. Specifically, we hypothesize that Si and B could influence enzyme activity by altering cellular redox balance or mitigating oxidative stress, although further investigations are warranted to confirm this hypothesis.

**Fig. 9:**
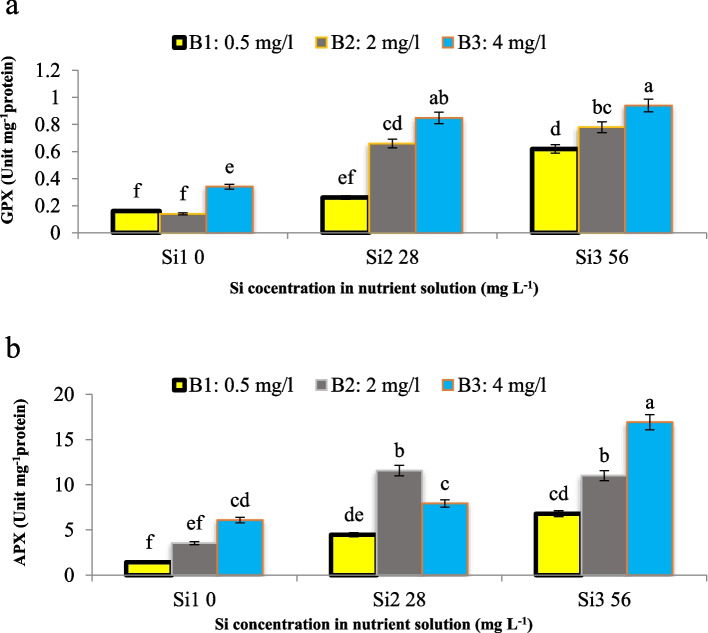
Interaction effect of different concentrations of Si and B on **a**) GPX and **b**) APX activity of maize var. Merit. Means not sharing the same letter do not differ significantly at *p* ≤ 0.01

Our study shed light on the intricate interplay between Si, B, and antioxidative enzyme activities, providing valuable insights into plant responses to environmental stresses. Thus, this investigation into the interaction between Si and B on the activities of GPX and APX revealed a significant effect (*p* ≤ 0.01). Our observations unveiled that an escalation in B concentration (4 mg L^−1^) under control conditions precipitated an augmentation in GPX activity. However, when Si application was combined with B, GPX activity surpassed that of the control conditions. Notably, the maximum GPX activity was achieved under the combined treatment of 4 mg L^−1^ B and 56 mg L^−1^ Si, as illustrated in Fig. 9a, while the lowest activity was observed in both the control and under 2 mg L^−1^ B concentration. Similarly, consistent results were observed in APX activity (Fig. 9b). Our findings resonate with previous studies, suggesting robustness and reliability in the observed trends [28, 29]. To explain these results comprehensively, we propose that the underlying mechanism driving the observed phenomenon lies in the synergistic or independent actions of Si and B on antioxidative enzyme activities. We hypothesize that Si and B may modulate the cellular redox balance, thereby influencing the activities of GPX and APX. Moreover, our assumption is supported by existing literature, which suggests that GPX serves as a biomarker for expressing the intensity of oxidative stress, particularly under conditions such as salinity and ion toxicity [28, 29]. Furthermore, GPX is known to be upregulated in response to various stressors including drought, cold, salt, gamma radiation, and heavy metal stress, indicating its role as a stress enzyme. GPX functions by catalyzing the decomposition of H_2_O_2_ into water and oxygen, effectively detoxifying and deactivating H_2_O_2_. On the other hand, APX, known for its high affinity for H_2_O_2_ compared to catalase, also scavenges H_2_O_2_ under stress conditions. The high affinity of APX for H_2_O_2_ stems from its unique catalytic mechanism, which involves the oxidation of ascorbate (vitamin C) as an electron donor to reduce H_2_O_2_ to water. This reaction is facilitated by the presence of a heme group in the active site of APX, which enables efficient binding and subsequent reduction of H_2_O_2_. Compared to catalase, APX exhibits a higher affinity for H_2_O_2_, particularly at lower concentrations, making it an effective scavenger of H_2_O_2_ under stress conditions where H_2_O_2_ levels are elevated. The increased activity of APX under abiotic stresses and heavy metal exposure further supports our findings, as it highlights the crucial role of this enzyme in detoxifying H_2_O_2_ and mitigating oxidative damage caused by reactive oxygen species.

### Minerals concentration

**Fig. 10:**
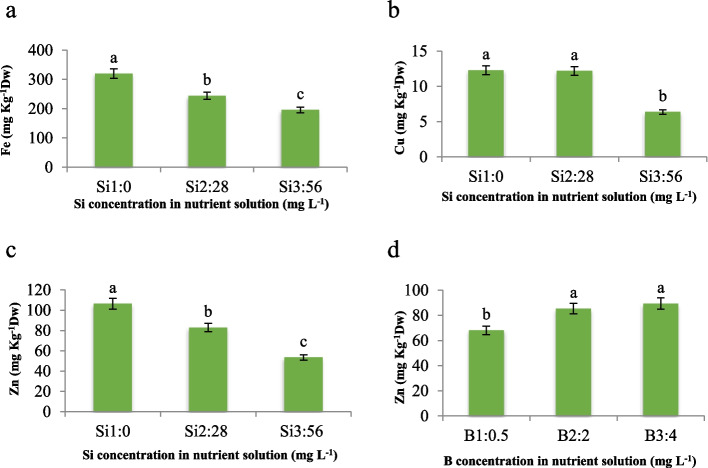
Effect of different concentrations of Si on **a**) Fe; **b**) Cu; **c**) Zn, and different concentrations of B in nutrient solution on **d**) Zn content of maize var. Merit**.** Means not sharing the same letter do not differ significantly at *p* ≤ 0.01

Our study explored the impact of Si and B on the concentrations of Fe, Cu, and Zn in maize leaves, aiming to elucidate the underlying mechanisms driving these effects. We found that Si application significantly influenced the concentrations of Fe, Cu, and Zn, whereas the effect of B was notable only on leaf Zn concentration. With increasing Si concentration, there was a marked decrease in the average Fe concentration in leaves, decreasing by 23.7% and 38.8% at Si concentrations of 28 and 56 mg L^−1^, respectively (Fig. 10a). Additionally, the use of 56 mg L^−1^ Si led to a significant 48% reduction in Cu content compared to the control, while no significant difference was observed with 28 mg L^−1^ Si (Fig. 10b). Furthermore, the addition of Si in the nutrient solution resulted in a decreasing trend in leaf Zn concentration, decreasing by 19.8% and 49.8% at Si concentrations of 28 and 56 mg L^−1^, respectively, compared to the control (Fig. 10c). Our findings are consistent with previous studies suggesting that Si can affect the absorption of various elements and ions crucial for chloroplast formation, such as Mg and Fe [30]. Similar results were observed in rice, where Si increased the oxidizing ability of the root, leading to the oxidation of Fe^+2^ to Fe^+3^ and preventing Fe absorption by forming Fe deposits [31]. Additionally, Si forms complexes with Cu, Zn, Mn, and other elements, reducing their transport through the symplast and forming unstable silicate compounds inside the cell. These silicates gradually decompose to produce SiO_2_, which remains in the cytoplasm, while the elements are transported into the vacuole and stored as complexes with organic acids [13]. Likewise, the increase in B concentration in the nutrient solution led to a significant increase in Zn concentration, approximately 1.3-fold higher than the control in both B concentrations (Fig. 10d). Zn is crucial as an essential micronutrient for plants, playing a vital role in the structure of proteins and enzymes, with higher mobility and transfer within the plant [32]. Our findings suggest that under hydroponic conditions, the absence of organic matter and clay particles may increase the availability of metals compared to soil solutions, potentially explaining the increased Zn absorption observed.

**Fig. 11:**
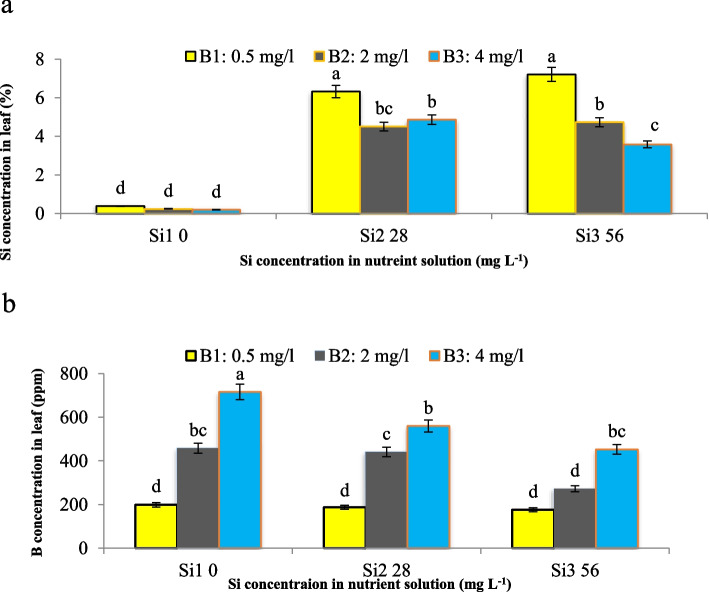
Interaction effect of different concentrations of Si and B on **a**) Si and **b**) B concentration in leaves of maize var. Merit. Means not sharing the same letter do not differ significantly at *p* ≤ 0.01

Our investigation examined the impact of Si and B interactions on the concentration of Si and B in maize leaves, aiming to unravel the underlying mechanisms driving these effects. We found a significant interaction effect of Si and B on Si concentration in maize leaves (*p* < 0.01). Interestingly, the highest leaf Si concentration was observed under control conditions, with both Si concentrations (28 and 56 mg L^−1^) exhibiting maximum Si levels in the absence of B toxicity (0.5 mg L^−1^ B). However, Si application mitigated B toxicity, evident from the reduced Si concentrations in leaves at Si concentrations of 28 and 56 mg L^−1^ (4.8% and 3.5% reduction, respectively) in the presence of 4 mg L^−1^ B (Fig. 11a). These findings align with previous studies by Gunes et al. [13, 19] in spinach and wheat, corroborating the consistent trend of increased Si concentration in response to Si application. Similarly, the interaction effect of Si and B in the nutrient solution significantly influenced B concentration in maize leaves (*P *< 0.05). Increasing Si concentration led to a notable reduction in leaf B concentration, decreasing by up to 21.7% and 36.7% at Si concentrations of 28 and 56 mg L^−1^, respectively, under severe B toxicity (4 mg L^−1^ B). In contrast, B toxicity resulted in increased B concentration in maize leaves in the absence of Si supplementation (Fig. 11b). This reduction of B in the presence of Si is likely attributed to the formation of borosilicate complexes in the soil or within the plant, which diminishes B availability [33]. The exogenous application of B in the nutrient solution typically precipitates an accumulation of B in the foliar tissues, whereas the concomitant application of Si and B attenuates B absorption due to the protective role of Si, consequently diminishing the concentration of B in leaves [34].

## Conclusion

Our findings from this study highlighted the significant impact of Si and B on various physiological and biochemical traits of maize plants. Increasing Si concentration in the nutrient solution led to enhanced plant growth, as evidenced by substantial increases in both fresh and dry weight, while B exhibited a detrimental effect, causing reductions in plant growth parameters. Importantly, Si application mitigated the adverse effects of B toxicity, indicating its potential role in promoting plant growth and counteracting B-induced stress. The study also revealed significant effects of Si and B on the concentrations of essential minerals such as Fe, Cu, and Zn in maize leaves, with Si reducing their absorption, potentially by forming complexes and altering their transport mechanisms. Furthermore, our findings shed light on the complex interplay between Si and B in regulating membrane stability, chlorophyll content, photosynthetic pigments, and antioxidative enzyme activities. Si supplementation proved effective in mitigating B-induced oxidative stress, enhancing antioxidant defense mechanisms, and improving overall plant health by increasing levels of total carbohydrate and total protein. These findings hold profound implications for the agricultural community, as they underscore the potential benefits of Si supplementation in optimizing plant growth, enhancing stress tolerance mechanisms, and ultimately augmenting crop yields, thereby offering a promising avenue for sustainable agricultural practices. Additionally, researchers can use these insights to further investigate the mechanistic underpinnings of Si and B interactions, conduct field trials to validate their efficacy under real-world agricultural conditions, and explore crop-specific responses to Si and B supplementation. To sum up, our study provided valuable insights into the potential of Si supplementation as a sustainable agronomic strategy to enhance crop productivity and resilience in the face of environmental stresses, thereby contributing to the advancement of agricultural sustainability and food security.

## Materials and methods

### Plant materials and treatments application

*Z. mays* var. Merit seeds (99% purity and 95% germination rate) were obtained from ASGROW company (USA) and were planted in plastic pots filled with sand. The plants were initially irrigated with modified Hoagland nutrient solution [35] (Table 1) until they reached the four-leaf stage. Subsequently, the plants were subjected to Hoagland nutrient solution supplemented with varying concentrations of Silicon (Si) (Na_2_SiO_3_.5H_2_O) at three levels (Si1: 0, Si2: 28, and Si3: 56 mg L^−1^) and B (H_3_BO_3_) at three concentrations (B1: 0.5, B2: 2, and B3: 4 mg L^−1^). The pH of the nutrient solution was adjusted to 6.5, and the electrical conductivity (EC) was monitored using the Aqualytic AL10con conductivity sensor, maintaining it at 1.55 dS m^−1^. For the control group, solely Hoagland solution devoid of any supplementary additives was used. Each plant was received approximately 1 L of the respective treatment solution three days a week. The temperature was maintained at 27 ± 3 °C during the day and 20 ± 3 °C during the night, with a relative humidity of about 60%. The experiment followed a factorial randomized complete block design with three replications, with two pots assigned to each treatment. Weekly rinsing of the plant substrate was conducted to prevent the accumulation of excess elements. After the final treatments, leaf samples were collected, immediately frozen in liquid nitrogen, and stored at -80 °C for subsequent physiological and biochemical analyses.

**Table 1:** Composition and concentration of salts in the modified Hoagland solution of Coolong et al. [35]

Nutrients	Concentration (mg L^−1^)	Nutrients	Concentration (g L^−1^)
H_3_BO_3_	2.86	Ca (NO_3_)_2_.4H_2_O	0.47
MnCl_2_.4H_2_O	1.81	KNO_3_	0.3
ZnSO_4_.7H_2_O	0.22	MgSO_4_. 7H_2_O	0.25
Na_2_MOO_4_.2H_2_O	0.02	NH_4_H_2_PO_4_	0.06
CuSO_4_.5H_2_O	0.08	FeEDTA	0.1

### Determination of growth characteristics

#### Fresh and dry weights of leaf

The sampled leaves were weighed freshly and then oven-dried at 80 ◦C for 48 h for measuring their dry weight.

### Determination of physiological characteristics

#### Membrane stability index (MSI)

The tubes containing leaf sample (0.1 g) and deionized water (10 mL) were heated at two different temperatures (40 ◦C for 30 min, C1 and 100 ◦C for 10 min, C2). Then, the tubes were cooled to room temperature and conductance measurements were made. Cell membrane stability was described as relative injury percentage and was calculated as follows [36]:

$$\mathrm{MSI}\;\left(\%\right)=1-\left(\mathrm C1/\mathrm C2\right)\times100$$

#### Chlorophyll index

The index of chlorophyll was quantified from the five fully expanded leaves from each pot utilizing a chlorophyll meter (Minolta, SPAD-504, Japan). The chlorophyll index of leaves was measured at 10-day intervals [37].

#### Photosynthesis pigments

Content of chlorophyll *a* (Chl *a*), chlorophyll *b* (Chl *b*), and chlorophyll *a* + *b* (Chl *a* + *b*) was detected using a spectrophotometer (UV-1800, Shimadzu, Japan) based on Arnon [38] assay. 0.5 g sampled leaves were homogenized with 80% acetone (1 mL) and were then centrifuged at 12,000 × g for 10 min (at 4 ◦C). The absorbance of supernatants was read at 470, 645, and 663 nm for Chl *a*, Chl *b*, and Chl *a* + *b* determinations, respectively.

### Determination of biochemical characteristics

#### Total soluble carbohydrates

The total soluble carbohydrate content was determined according to the DuBois et al. [39] method. The powder leaf samples (0.5 g) were homogenized with 80% ethanol (1.5 mL). Then, the mixtures were centrifuged at 5000 × g for 10 min and the supernatants were placed into the water bath at 50 ◦C to removing ethanol from samples. After that, 10 mL of water, 0.47 mL of 0.3 N hohydroxide barium (BaH_2_O_2_), and 0.5 mL of 5% zinc sulfate (ZnSO_4_) were added to the samples and were then centrifuged (5000 × g for 10 min). At that time, 1 mL of the supernatants were mixed with 0.5 mL of 5% phenol and 2.5 mL of 98% sulfuric acid and were placed in the dark for 45 min. The absorbance of samples was read at 485 nm using the spectrophotometer.

#### Total soluble protein

The leaf sample (0.5 g) was homogenized with a 1.5 mL of potassium phosphate buffer (K_2_SO_4_, pH = 7) and 0.025 g of polyvinylpolypyrrolidone (PVPP). The extracts were centrifuged at 15,000 rpm for 30 min and supernatants were used for determining total soluble protein and activity of antioxidant enzymes. Total soluble protein was measured based on Bradford [40] assay at 595 nm.

#### Hydrogen peroxide (H_2_O_2_)

 Determination of the H_2_O_2_ content in maize was carried out following the protocol of Liu et al. [41]. So, 0.5 g samples of rose petals were ground in liquid nitrogen and a potassium phosphate buffer (pH 6.8). The extracts of the distinct samples were centrifuged at 7000 rpm for 25 min at 4 °C. A 100 µL aliquot of the supernatants was added to 1 mL of xylenol solution, mixed completely and allowed to breathe for 30 min. Then the extent of absorbance was measured by spectrophotometer (UV-1800, Shimadzu, Japan) at 560 nm.

#### Malondialdehyde (MDA)

 The content of Malondialdehyde (MDA), as a product of lipids peroxidation, was measured based on assay of Zhang et al. [42]. The powder leaf sample (0.1 g) was homogenized with 1.5 mL of 1% Trichloroacetic acid (TCA) and the extract was centrifuged at 10,000 × g at 4 ◦C for 10 min. then, 1 mL of 0.5% Thiobarbituric acid (TBA) and 20% TCA was added to the supernatants. The mixtures were place into water bath at 95 ◦C for 30 min. the absorbance of supernatants was read at 52 and 600 nm and an extinction coefficient 155 mM^−1^ cm^−1^ was considered for calculation of MDA activity.

#### Catalase (CAT)

 The activity of Catalase (CAT) was determined according to Aebi [43] method. The mixture reaction included 750 µL of 25 M K_2_SO_4_ buffer (pH = 7), 750 µL of 10 mM hydrogen peroxide (H_2_O_2_), and 20 µL of protein extract. The activity of CAT was calculated based on an extinction coefficient 39.4 mM^−1^ cm^−1^ for H_2_O_2_ at 240 nm.

#### Guaiacol peroxidase (GPX)

For determining the activity of guaiacol peroxidase (GPX), the absorbance of the mixture reaction (containing 750 µL of 100 mM K_2_SO_4_ buffer (pH = 7), 100 µL of 70 mM H_2_O_2_, and 750 µL of 10 mM guaiacol) was read at 470 nm [44]. An extinction coefficient 26.6 mM^−1^ cm^−1^ for guaiacol was considered for calculation of GPX activity.

#### Ascorbate peroxidase (APX)

Ascorbate peroxidase activity (APX) was measured according to the Nakano and Asada [45] (1981) method with some slight changes. The mixture reaction for determining APX was 200 µl of 2 mM ascorbate dissolved in 100 mM K_2_SO_4_ buffer (pH = 7), 200 µL of 10 mM H_2_O_2_, and 30 µL of 5 mM Ethylenediaminetetraacetic acid (EDTA), and 20 µL of protein extract. The absorbance of the mixture reaction was read at 290 nm and the activity of APX was calculated using an extinction coefficient 2.8 mM^−1^ cm^−1^.

### Content of minerals

Minerals concentration was measured by the wet digestion [46]. The leaf samples were washed with deionized water and air-dried. Then, the leaves were dried in an oven at 550 ◦C for 6 h. After cooling to room temperature, 10 mL of 65% HNO_3_ was added to the inorganic residue in the crucibles and they were placed in the digester without heating for 1 day. On the subsequent day, the samples underwent a two-step heating process, initially at 65 °C for a duration of 3 h, followed by a higher temperature of 110 °C for an additional 3-h period. The final clear solutions were filtered with Whatman paper N.42 and were transferred to a 100 mL volumetric flask and volume was made up with deionized water. Fe, Zn and Cu were determined directly in final digests using an atomic absorption spectrophotometry (UV-1800, Shimadzu, Japan). For the determination of Si, the leaf samples were homogenized with 50 mL of 0.08% H_2_SO_4_ and 2 mL of 40% HF. A solution including 1.5 mL of 0.08% H_2_SO_4_, 20 g L^−1^ of (NH_4_)_6_Mo_7_O_24_ and 1.5 mL of 0.2 M C_4_H_6_O_6_ were added to the 1.5 mL of extract. The absorbance of the obtained solution was read at 811 nm [47]. The content of B was determined by colorimetric assay using Azomethine-H reagent. This reagent was prepared by dissolving 0.45 g of Azomethine-H in 100 mL of 1% L-ascorbic acid. Acetate buffer solution was ready by dissolving 25 g of ammonium acetate and 15 g of Na_2_-EDTA in 400 mL of deionized water and then 125 mL of acetic acid was added to it. A 0.36 N H_2_SO_4_ was used for the extraction of samples. The extracts were filtered w and transferred to a 50 mL volumetric flask and volume was made up with deionized water. B standard solution was prepared by dissolving 0.114 g of boric acid in 1 L of deionized water for preparing the concentrations of 0, 0.5, 1, 1.5, 2, 2.5 mg L^−1^. The absorbance of plant samples and standards was read at 420 nm [48].

### Statistics

The factorial experiment was carried out according to a completely randomized block design with 3 replications. Data for parameters were statistically analyzed by MSTAT-C ver 2.1 software and means were separated using the Duncan the levels of five and one percent error probability.

## Data Availability

Correspondence and requests for materials should be addressed to H.S.H.
